# Excessive Autophagy Activation and Increased Apoptosis Are Associated with Palmitic Acid-Induced Cardiomyocyte Insulin Resistance

**DOI:** 10.1155/2017/2376893

**Published:** 2017-11-26

**Authors:** Shanxin Li, Hui Li, Di Yang, Xiuyan Yu, David M. Irwin, Gang Niu, Huanran Tan

**Affiliations:** ^1^Department of Pharmacology, School of Basic Medical Sciences, Health Science Center, Peking University, Beijing 100191, China; ^2^Department of Laboratory Medicine and Pathobiology, University of Toronto, Toronto, ON, Canada M5S 1A8; ^3^Beijing N&N Genetech Company, Beijing 100082, China

## Abstract

Diabetic cardiomyopathy (DCM) remains the major cause of death associated with diabetes. Researchers have demonstrated the importance of impaired cardiac insulin signaling in this process. Insulin resistance (IR) is an important predictor of DCM. Previous studies examining the dynamic changes in autophagy during IR have yielded inconsistent results. This study aimed to investigate the dynamic changes in autophagy and apoptosis in the rat H9c2 cardiomyocyte IR model. H9c2 cells were treated with 500 *μ*M palmitic acid (PA) for 24 hours, resulting in the induction of IR. To examine autophagy, monodansylcadaverine staining, GFP-LC3 puncta confocal observation, and Western blot analysis of LC3I-to-LC3II conversion were used. Results of these studies showed that autophagic acid vesicles increased in numbers during the first 24 hours and then decreased by 36 hours after PA treatment. Western blot analysis showed that treatment of H9c2 cells with 500 *μ*M PA for 24 hours decreased the expression of Atg12-Atg5, Atg16L1, Atg3, and PI3Kp85. Annexin V/PI flow cytometry revealed that PA exposure for 24 hours increased the rate of apoptosis. Together, this study demonstrates that PA induces IR in H9c2 cells and that this process is accompanied by excessive activation of autophagy and increases in apoptosis.

## 1. Introduction

It was recently estimated that 415 million people suffered from diabetes globally in 2015 [1]. Diabetic cardiomyopathy (DCM) is a major complication that accounts for more than half of the diabetes-related morbidity and mortality cases [2]. DCM has been defined as ventricular dysfunctions that occur in diabetic patients independent of recognized cause, such as coronary artery disease or hypertension [3]. Insulin resistance, which is defined by a decrease in glucose disposal in response to insulin by target tissues [4], is an important risk factor for cardiovascular morbidity [4, 5]. However, the molecular mechanisms of insulin resistance in the pathogenesis of DCM remain to be elucidated.

Elevated free fatty acid (FFA) plasma levels contribute to obesity-associated insulin resistance. Previous research demonstrated that increased lipid levels in the heart result in deficiencies of myocardium contraction and left ventricular dysfunction [6]. Saturated long-chain FFA such as palmitic acid (PA) and stearic acid was potent inducers of these dysfunctional effects [7]. PA is the main component of dietary saturated fat, accounting for nearly 20% of the total serum FFA. High levels of PA are widely used to study FFA-induced insulin resistance [8]. In vitro studies indicated that insulin resistance was induced in a human endothelial cell line by 250 to 1000 *μ*M PA [9] and in primary rat ventricular myocytes by 500 *μ*M PA for 24 hours [10]. In vivo experiments showed that high levels of palmitic acid lead to insulin resistance due to changes in the level of phosphorylation of the insulin receptor and insulin receptor substrate-1 in rats [4].

Autophagy is a cellular protein degradation system that enables cells to recycle cytoplasmic components by degradation in the lysosomes [11]. While there have been a few studies examining the relationship between autophagy and insulin resistance, their conclusions have been inconsistent. Yang et al. [12] observed a downregulation of autophagy, particularly in Atg7 expression levels, in both genetic and dietary models of obesity. They found that the suppression of Atg7, both in vitro and in vivo, resulted in defective insulin signaling. In contrast, Ost et al. [13] demonstrated attenuated mTOR signaling and enhanced autophagy in adipocytes from obese patients with type 2 diabetes. Rapamycin (RAP) induces autophagy by inhibiting mammalian target of rapamycin complex-1 (MTORC1) [14] and is routinely applied in the study of autophagy regulation; thus, we used RAP as a positive control for autophagy induction in our study [15, 16].

We hypothesize that basal levels of autophagy are necessary for maintaining cellular insulin signaling, yet excessive autophagy may lead to impaired insulin signaling. The aim of the present study was to explore the role of autophagy in insulin resistance in rat cardiomyocyte model induced by PA to elucidate the molecular mechanisms leading to DCM and help find candidate drug targets to treat DCM.

## 2. Materials and Methods

### 2.1. Materials and Reagents

Dulbecco's Modified Eagle Medium (DMEM) culture media were obtained from Life Technologies (USA). Autophagy inducer rapamycin (RAP) and 4′,6′-diamidino-2-phenylindole (DAPI) were purchased from Solarbio (Beijing, China). Autophagy inhibitor hydroxychloroquine sulfate (HCQ) was obtained from Tokyo Chemical Industry (Japan). Monodansylcadaverine (MDC) was purchased from Sigma-Aldrich LLC (USA).

### 2.2. Cell Culture

Immortalized rat cardiac myoblastic cells H9c2 were obtained from the Shanghai Institute of Biochemistry and Cell Biology (China). Cells were cultured in high-glucose DMEM medium supplemented with 100 U/ml penicillin, 100 *μ*g/ml streptomycin, and 10% heat-inactivated FBS (Thermo Fisher Scientific, USA) and maintained at 37°C and 5% CO_2_. One day before experiments, cells were incubated in culture medium supplemented with 1% FBS to allow the cells to differentiate into cardiomyocytes [17].

### 2.3. PA Treatment

PA was administered to cells by conjugating it with bovine serum albumin (BSA) as described by Chavez et al. [18]. Briefly, PA was completely dissolved in 100% ethanol and diluted 1 : 100 in 1% FBS-DMEM containing 2% (*w*/*v*) bovine serum albumin. The control treatment was prepared by adding the same amount of ethanol to BSA-DMEM solution. All solutions were filtered, aliquoted, and stored at −20°C prior to use.

### 2.4. Measurement of Glucose Consumption

The consumption of glucose was measured as previously described [17, 19]. H9c2 cells were seeded into 96-well culture plates (5000 cells/well) in 100 *μ*l medium for 24 h. The medium was then replaced with media with 0, 250, or 500 *μ*M PA with or without 100 nM RAP, and cells were cultured for another 12 or 24 h. Insulin (100 nM) was then added to each well for the last 30 minutes. Glucose was measured from 8 *μ*l of media removed at 12 and 24 h of culture. The concentration of glucose in the cell media was measured with blood glucose test paper (Roche, Switzerland), with glucose production calculated after subtracting the glucose concentration found in media from DMEM culture medium wells without cells.

### 2.5. Western Blot Analysis

Total protein was extracted using a lysis buffer containing 50 mM Tris–HCl (pH 8.0), 150 mM NaCl, 0.02% NaN_3_, 0.1% SDS, 1 mM EDTA, 1% Triton X-100, and 100 mg/ml PMSF. For each sample, 50 *μ*g of protein was separated by SDS-PAGE (with different concentrations appropriate for the molecular weight of the targeted proteins) at 80 V for 0.5 h and 120 V for 1 h using the Mini-PROTEAN 3 electrophoresis cell system (Bio-Rad, USA). Proteins were then transferred to a PVDF membrane (Bio-Rad, USA) by the semidry blotting method with Dunn carbonate transfer buffer consisting of NaHCO_3_ (10 mM), Na_2_CO_3_ (3 mM), and 20% methanol. Membranes were then treated with 5% nonfat milk powder in 1XTBST buffer for 1 h to block nonspecific binding and then incubated overnight at 4°C with primary antibodies. Antibodies were rabbit anti-IR-*β*, IRS2, LC3I and II, Atg3, Atg12, Atg16L1, Bcl2, PI3Kp85 (1 : 500, CST, USA), and mouse anti-GAPDH (1 : 1000, Abcam, USA). Antibody binding was detected using goat anti-rabbit or goat anti-mouse secondary antibodies (ZSGB Bio, China), which were added for 1 h, washed 3 times, and then visualized by luminal chemiluminescence ChemiDoc XRS (Bio-Rad, USA). Band intensities were semiquantitatively analyzed by quantity-one software.

### 2.6. Detection of Autophagic Vesicles by Monodansylcadaverine Staining

Monodansylcadaverine (MDC), a fluorescent compound, was used as a tracer of autophagic vacuoles [20]. H9c2 cells were incubated with 50 *μ*M MDC for 30 min at 37°C, according to preview literature's method [21]. Cells were then washed twice with PBS and fixed with 4% paraformaldehyde for 15 minutes before autophagosomes being observed with a confocal microscope (excitation spectrum. 405 nm), (Leica TCS SP8, Germany). Data analysis was performed using Image-Pro Plus 6.0 (Media Cybernetics, USA), and the MDC positive area was calculated for each visual field.

### 2.7. Detection of Autophagy Flow by GFP-LC3

Autophagosomes were labeled with GFP to detect their movement. H9c2 cells were transfected with a lentivirus plasmid vector containing GFP-LC3 (a gift from Qi-hua He, Center of medical and health analysis, Peking University) that targets autophagosomes. Transfected cells were seeded onto confocal culture dishes and treated with 500 *μ*M PA, with or without 100 nM RAP for 24 h and with or without 50 *μ*M HCQ. HCQ was added for only the last 2 hours. Cells were then fixed with 4% paraformaldehyde and washed 3 times with PBS. Fixed cells were stained with DAPI for 15 minutes, washed 3 times with PBS, and observed with a confocal microscope (GFP excitation spectrum: 488 nm; DAPI excitation spectrum: 405 nm) (Leica TCS SP8, Germany). To assay autophagic flow, “LC3 net flux” was calculated after subtraction of the amount of LC3II in the absence of HCQ from the amount of LC3II in the presence of HCQ for each condition [22]. Data analysis was performed using Image-Pro Plus 6.0 (Media Cybernetics, USA) and calculated for GFP-LC3 area per cell.

### 2.8. RNA Extraction and Real-Time PCR

Total mRNA was extracted from H9c2 cells using TRIzol reagent (Invitrogen, USA). After the concentration of the purified mRNA was verified, reverse transcription was conducted using RevertAid first Strand cDNA Synthesis Kit (Thermo Scientific, USA) according to the manufacturer's instructions. Expression of target genes was measured by semiquantitative RT-PCR using BrilliantII SYBR Green QPCR Master Mix (Agilent, USA) and gene-specific primers following the manufacturer's protocol. PCR reaction conditions were as follows: initial denaturation at 95°C for 10 min and then 40 cycles of denaturation at 95°C for 30 s and annealing/extension at 60°C for 30 s, 72°C for 30 s. Cycle number at threshold (Ct value) was used to calculate the relative amount of the mRNA molecules and is presented as fold-change compared to *β*-actin, calculated using the 2^△△Ct^ method [23]. Gene-specific primer sequences used for RT-PCR were as follows:

Beclin1 F: 5′-AGATGCGCTATGCCCAGATG-3′, R: 5′-AATTGTCCGCTGTGCCAGAT-3′;

mTOR F: 5′-CACCCATCCAACCTGATGCT-3′, R: 5′-TCGAGACCGGTAACCTCCAT-3′;

*β*-actin F: 5′-TACAACCTTCTTGCAGCTCCT-3′, R: 5′-TGACCCATACCCACCATCAC-3′.

### 2.9. Cell Viability

Cell viability was examined using the MTS assay with CellTiter 96® AQueous One Solution (Promega Biotech, USA) according to the manufacturer's instructions. Briefly, H9c2 cells were seeded into 96-well culture plates (5000 cells/well) with 100 *μ*l medium and incubated overnight to allow the cells to adhere. Cells were then treated with PA (0, 250, and 500 *μ*M) with or without nM RAP for 24 h. For the last 4 hours of culture, 20 *μ*l of MTS solution was added to each well. Absorbance was measured at 490 nm using an iMarK microplate reader (Bio-Rad, USA).

### 2.10. Apoptosis Determination

Cellular apoptosis was determined using the StarGlow Annexin V-FITC Apoptosis Detection Kit (Genestar, China). Briefly, 1 × 10^6^ cells from different treatments were rinsed with PBS and suspended with 100 *μ*l of binding buffer. Subsequently, 5 *μ*l Annexin V-FITC was added and incubated for 5 min under dark conditions, and 10 *μ*l PI was added before flow cytometry (BD Biosciences, USA).

### 2.11. Statistical Analysis

Data are presented as mean ± standard error of mean (SEM) from three independent experiments. Statistical differences were assessed using one-way ANOVA. First, we checked whether the variances of the data were homogeneous. If variances were homogeneous, then the LSD statistics were applied as the post hoc test, if not, then the Tamhane statistics was employed. *P* values less than 0.05 were considered to be significant.

## 3. Results

### 3.1. Insulin Resistance in H9c2 Cells Induced by 500 *μ*M PA Treatment for 24 h

**Figure 1:**
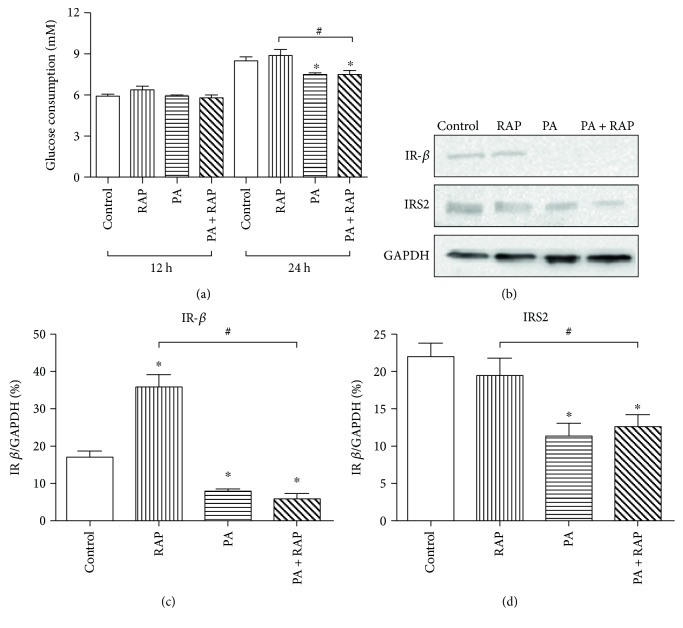
Insulin resistance in H9c2 cells was induced by 500 *μ*M PA treatment for 24 h. (a) H9c2 were treated with solvent (control), 100 nM RAP (RAP), 500 *μ*M PA (PA), 500 *μ*M PA+ 100 nM RAP (PA + RAP), and cultured for 12 h and 24 h. Cells were incubated by adding 100 nM insulin for the last 30 minutes. Glucose consumptions were measured. H9c2 cells were treated with drugs for 24 h and 100 nM insulin was then added for the last 10 minutes. (b) Western blot analysis of IR-*β* and IRS2 compared with GAPDH. (c, d) Quantification of the results from (b) (*n* = 3). ^∗^*P* < 0.05 compared with the controls of the same time period. ^#^*P* < 0.05 compared with RAP groups of the same time period.

To induce insulin resistance model, H9c2 cells were treated with 500 *μ*M PA with or without RAP for 12 and 24 hours [10], and glucose consumption was measured. No changes in glucose consumption were found among groups after 12 hours (Figure 1(a), *P* > 0.05). Glucose consumptions of the 500 *μ*M PA and the 500 *μ*M PA + RAP groups were less than the controls at 24 h (Figure 1(a), *P* < 0.05), with the glucose consumption of the 500 *μ*M PA + RAP group being less than that of the RAP group (Figure 1(a), *P* < 0.05). H9c2 cells treated with lower PA concentrations (250 *μ*M) for 24 hours did not show any decrease in glucose consumption compared to the control cells (data not shown).

Protein markers for insulin resistance were examined after 24 h of treatment groups. Expression of IR-*β* was inhibited in cells treated with 500 *μ*M PA compared with the control group (Figures 1(b) and 1(c), *P* < 0.05), with the levels of IR-*β* in the 500 *μ*M PA + RAP group significantly lower than those in the RAP and control groups (Figures 1(b) and 1(c), *P* < 0.05). Similar changes were seen for IRS2 (Figures 1(c) and 1(d), *P* < 0.05). Together, these data indicated that H9c2 cells acquired IR after exposure to 500 *μ*M PA for 24 h.

### 3.2. PA-Augmented Activation of Autophagy

**Figure 2:**
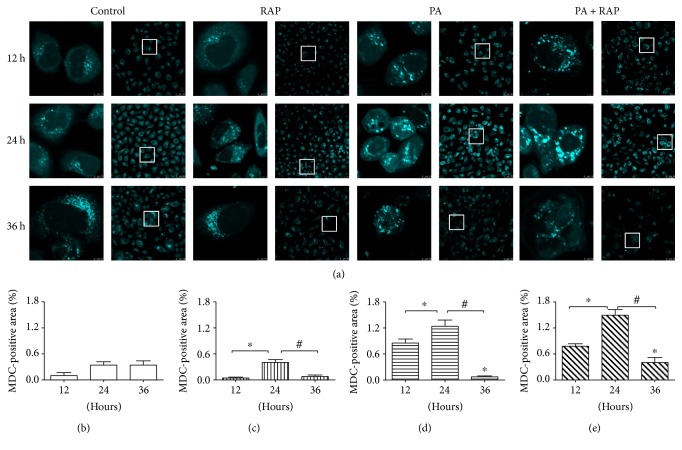
PA-augmented activation of autophagy flow as evidenced by MDC fluorescence. (a) H9c2 cells were treated as in Figure 1 and cultured for 12, 24, and 36 h. Cells were stained with 50 *μ*M MDC for 30 minutes. MDC-positive area per visual field was counted in (b) for the control, (c) for RAP, (d) for PA, and (e) for PA + RAP group (*n* = 5). ^∗^*P* < 0.05 compared with the 12 hour group. ^#^*P* < 0.05 compared with the 24 hour group.

The role of autophagy during insulin resistance has been controversial [13, 24, 25]; thus, we employed multiple techniques to demonstrate that PA induces dysfunctional autophagy. As autophagy is a dynamic process, we applied MDC, which accumulates in acidic vesicles, for the detection of autophagosome formation. The size of the MDC-positive particle area in cells of the 500 *μ*M PA group was increased at 24 h compared to 12 h and then decreased at 36 h (Figures 2(a) and 2(d), *P* < 0.05). A similar trend was seen for the RAP and 500 *μ*M PA + RAP groups (Figures 2(a), 2(c), and 2(e), *P* < 0.05). However, the MDC-positive particle area did not change in the control groups (Figures 2(a) and 2(b), *P* > 0.05). Based on these findings, we hypothesized that PA enhanced autophagy flow for the first 24 hours and then decreased by 36 hours, a pattern similar to that seen with rapamycin.

**Figure 3:**
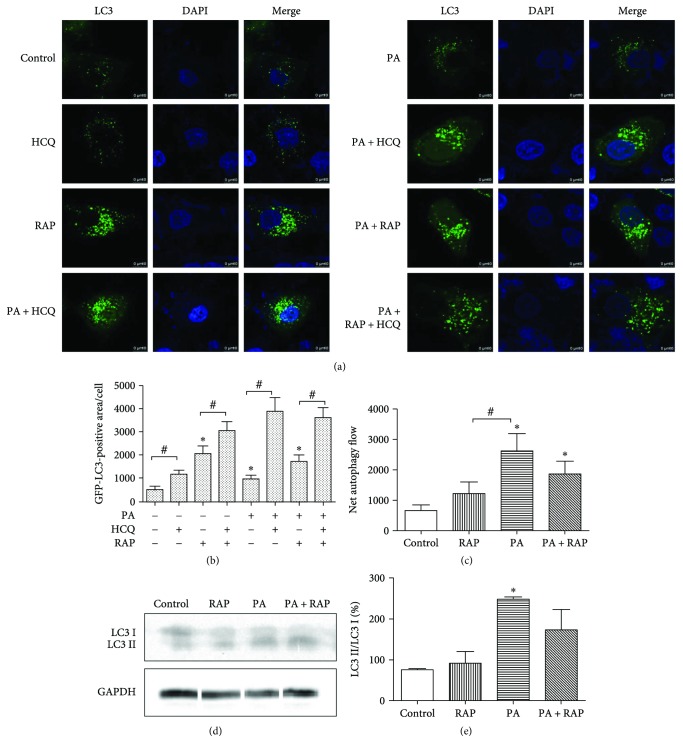
PA did not block autophagy, as evidenced by GFP-LC3 fluorescence and LC3 conversion. (a) H9c2 was treated with or without 500 *μ*M PA, 100 nM RAP, and cultured for 24 h. HCQ (50 *μ*M) was added for the last 2 hours. GFP-LC3 puncta were visualized by confocal microscopy. (b) GFP-LC3 puncta area per cell was measured from 10 random visual fields. (c) “LC3 net flux” was calculated for each of the treatments (*n* = 10). (d, e) H9c2 cells were treated as in Figure 1 for 24 h, and LC3II/LC3I levels were semiquantitated by Western blot and compared with GAPDH (*n* = 3). ^∗^*P* < 0.05 compared with the control. ^#^*P* < 0.05 compared with the arrow-headed group.

We then wondered whether the increase in the number of autophagosomes was due to enhanced autophagic activity or blockage of autophagosome fusion with lysosomes. To study this, we used assays with GFP-LC3. GFP-LC3 lentivirus was employed to transfect H9c2 cells, generating cells that stably express GFP-LC3. The lysosomal inhibitor HCQ (50 *μ*M) was used to block LC3II fusion with lysosomes [22]. As illustrated in Figures 3(a) and 3(b), 500 *μ*M PA or RAP increased GFP-LC3 puncta per cell, and HCQ treatment in combination with PA or RAP yielded a significantly increased GFP-LC3 puncta (Figures 3(a) and 3(b), *P* < 0.05). Based on these results, we speculated that increased number of autophagosomes seen with PA exposure was mostly attributed to excessive activation of autophagic initiation, rather than the blockage of fusion of autophagosomes with lysosomes.

To measure the amount of fusion of autophagosomes (represented by LC3 puncta) with lysosomes, we defined the concept “LC3 net flux,” which is calculated by subtracting the average amount of GFP-LC3 in the absence of HCQ from the amount of GFP-LC3 in the presence of HCQ for each condition [22]. Our results showed that LC3 net flux in H9c2 cells was enhanced upon exposure to 500 *μ*M PA with or without RAP for 24 hours compared to the control group (Figures 3(a) and 3(c), *P* < 0.05). This suggests that, compared with the control group, treatment with 500 *μ*M PA for 24 hours resulted in excessive autophagy flow. The ratio of LC3II/LC3I in 500 *μ*M PA-treated cells was significantly higher than in the control cells (Figures 3(d) and 3(e), *P* < 0.05), which indicates that 500 *μ*M PA increased the conversion of LC3I to LC3II. As an increase of LC3 conversion means enhanced autophagy flow [22], this supported the conclusion that 500 *μ*M PA augmented autophagy.

### 3.3. PA Decreased Remaining Autophagy-Related-Gene Proteins at 24 h

**Figure 4:**
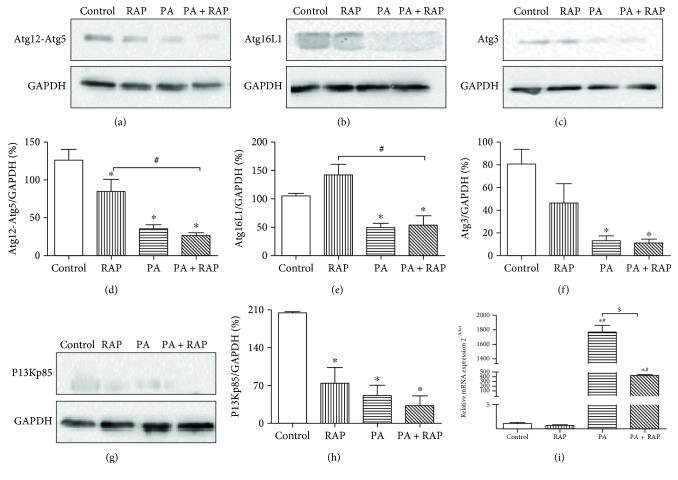
Changes in Atg and PI3K proteins and mTOR mRNA after PA treatment for 24 h. (a–h) H9c2 was treated as in Figure 1 for 24 h. Protein levels of Atg12-Atg5, Atg16L1, Atg3, and PI3Kp85 were semiquantitated by Western blot compared with GAPDH. (*n* = 3). (i) H9c2 cells were treated as in Figure 1 for 24 h, and the mRNA of levels mTOR were measured by semiquantitative RT-PCR, with *β*-actin as the control. (*n* = 3). ^∗^*P* < 0.05 compared with the control. ^#^*P* < 0.05 compared with the RAP group. ^$^*P* < 0.05 compared with the PA + RAP group.

In addition to the LC3 analysis, we also measured the protein expression levels of autophagy-related-genes (Atg) by Western blot after 24 h treatment. Exposure to 500 *μ*M PA with or without RAP caused a larger decrease in protein levels of Atg12-Atg5 compared to the control (Figures 4(a) and 4(d), *P* < 0.05). Also, 500 *μ*M PA + RAP group showed significantly lower level of Atg12-Atg5 compared with the RAP group (Figures 4(a) and 4(d), *P* < 0.05). Since Atg12 combines with Atg5 soon after it is synthesized [26], we did not detect free Atg12 (data not shown). Atg16L1 binds to Atg12-Atg5 to form a homodimer, and Atg12-Atg5-Atg16L1 dimers are important for LC3-PE conjugation [26]. Western data showed that 500 *μ*M PA led to significantly decreased Atg16L1 levels (Figures 4(b) and 4(e), *P* < 0.05). Atg3 is a protein necessary for LC3 activation [26]. The protein levels of Atg3 in the 500 *μ*M PA and 500 *μ*M PA + RAP groups were significantly lower than in the controls (Figures 4(c) and 4(f), *P* < 0.05), which was consistent with the levels of Atg12-Atg5 and Atg16L1. These results, together with MDC and LC3 analysis, imply that the decreases of Atg proteins in the 500 *μ*M PA-treated cells are due to excessive autophagic activation.

### 3.4. PI3K/Akt/mTOR Pathway Was Involved in PA-Induced IR and Dysregulated Autophagy

Does pathway upstream of autophagy change in the H9c2 IR model? To examine this, we measured the protein levels of the PI3Kp85 subunits and the mRNA levels for mTOR and Akt. The protein levels of the PI3Kp85 subunit in the RAP, 500 *μ*M PA, and 500 *μ*M PA + RAP groups were lower than that in the control (Figures 4(g) and 4(h), *P* < 0.05). The mTOR mRNA in 500 *μ*M PA and 500 *μ*M PA + RAP-treated cells were higher than that in the controls (Figure 4(i), *P* < 0.05). No significant changes in the mRNA levels of Akt were observed (data not shown).

### 3.5. 500 *μ*M Palmitic Acid Reduced Viability and Increased Apoptosis of H9c2 Cells

**Figure 5:**
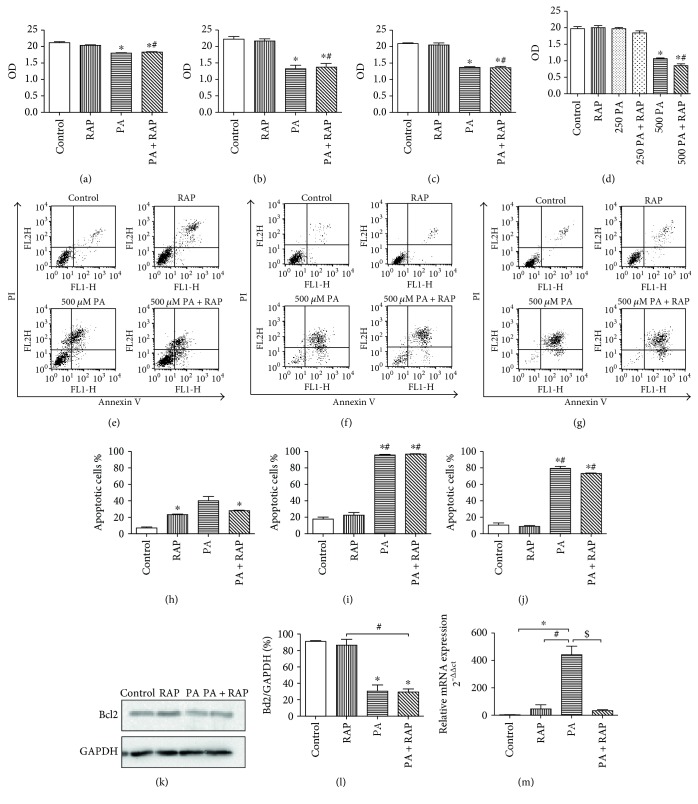
Increased apoptosis of H9c2 cells treated with 500 *μ*M PA. (a–d) H9c2 cells were treated with 250 *μ*M and 500 *μ*M PA, cultured for 24 h, and viability was measured by the MTS assay. (e–j) Changes in the rate of apoptosis with PA treatment. H9c2 were treated as in Figure 1 and cultured for 12 h (e, h), 24 h (f, i), and 36 h (g, j). Annexin V/PI staining flow cytometry was used to quantify the proportion of cells undergoing apoptosis. (e–g) Typical graphs for each time points. (h–j) Quantification of the flow cytometry results. Percentage of apoptotic cells is the sum of the events in the upper right and lower right quadrants. (k, l) H9c2 were treated as in Figure 1 for 24 h, and Bcl2 protein was semiquantitated by Western blot, with GAPDH as the loading control (*n* = 3). (m) mRNA levels of Beclin1 were measured by semiquantitative RT-PCR with *β*-actin as the control (*n* = 3). ^∗^*P* < 0.05 compared with the control. ^#^*P* < 0.05 compared with the RAP groups. ^$^*P* < 0.05 compared with the PA + RAP group.

We next explored whether PA influenced cell viability and apoptosis in the H9c2 insulin resistance model. H9c2 cells were exposed to 500 *μ*M PA with or without 100 nM RAP for 12, 24, and 36 hours, and cell viability was measured using MTS. Our results revealed that 500 *μ*M PA reduced the viability of H9c2 cells compared with the control (Figures 5(a) and 5(c), *P* < 0.05) at all of the time points, whereas, cell viability in the 250 *μ*M PA group at 24 h did not change (Figure 5(d), *P* > 0.05).

To understand the mechanisms underlying PA-induced cytotoxicity of H9c2 cells, we explored the role of apoptosis after treatment of H9c2 cells with PA. An Annexin V/PI analysis was used to measure apoptosis. Our data showed that the rate of apoptosis in 500 *μ*M PA-treated cells did not change at 12 hours (Figures 5(e) and 5(h), *P* > 0.05) but was significantly increased at 24 h and 36 h compared with the control (Figures 5(f), 5(g), 5(h), 5(i), and 5(j), *P* < 0.05). These results suggest that 500 *μ*M PA increases the level of apoptosis during the development of H9c2 insulin resistance. Bcl2 and beclin1 are key proteins for the interactions between apoptosis and autophagy [27] As a BH3-only Bcl2 family protein, Beclin1 is regulated by Bcl2 family proteins through its BH3 domain [28]. Treatment of H9c2 cells with 500 *μ*M PA for 24 hours decreased the protein levels of Bcl2 (Figures 5(k) and 5(l), *P* < 0.05) while the mRNA levels of Beclin1 mRNA were significantly higher than in the three other groups (Figure 5(m), *P* < 0.05).

## 4. Discussions

Several factors have been proposed to cause IR, including inflammation, mitochondrial dysfunction, hyperinsulinemia, hyperlipidemia, and aging [3]. Many of these factors are associated with obesity, which is the major risk factor for IR in the general population [29]. Lipotoxicity induced by high concentrations of circulating FFA is a key mechanism of IR development, and among these FFAs, PA is the most common [30]. Several studies have revealed that PA influences autophagy, but their conclusions were controversial [13, 24]. In the present study, we addressed the effects of PA on autophagy in IR cardiomyocytes.

First, we established a cardiomyocyte IR model by exposing H9c2 to 500 *μ*M PA for 24 hours, as Cao et al. showed that insulin resistance effect was induced in primary rat ventricular myocytes treated with 500 *μ*M PA [10]. The cell model was validated by inhibition of glucose consumption and decreases in the protein levels for IR-*β* and IRS2. IR-*β* tyrosine kinase, when activated by insulin, phosphorylates IRS proteins [31]. IRS1 and IRS2 then activate the PI3K/Akt pathway. Studies have shown that IRS2 polymorphisms are related to insulin resistance, thus IR-*β* and IRS2 are useful markers for the detection of insulin resistance [32]. Our results were similar with those from previous studies. For example, murine C2C12 myotubes and human umbilical vein endothelial cells (HUVECs) [33] required 750 *μ*M and 100 *μ*M PA, respectively, to induce IR. As these cells are from different species and tissues, the cellular metabolism is likely not the same; thus, differing levels of PA lead to IR. PA-induced IR is also observed in animal models. Battiprolu et al. [34] showed that mice develop myocardial IR in response to high fat-diet, where it is characterized by downregulation of IR activity, decreased Akt signaling, and a shift from glucose to fatty acid utilization. Therefore, we established a cardiomyocyte IR by saturated fatty acid.

Autophagy is a lysosomal degradation process through which misfolded proteins and organelles are sequestered, degraded by lysosomes, and recycled [15]. Autophagy is an essential part of cardiomyocyte homeostasis and increases the survival of cells following cellular stress and starvation [35]. Under conditions of nutrient deprivation, induction of autophagy provides cells with an opportunity to reutilize their own constituents for energy [22]. However, under specific circumstances, autophagy not only protects cells against death but also mediates cell death. The morphological features of autophagy, which are distinct from apoptosis, have been observed in dying cells. If autophagy destroys cytosol and organelles beyond a certain threshold, then autophagic cell death will occur [36]. As glucose uptake is impaired in tissues with insulin resistance, we investigate whether autophagy changed in response to IR in our H9c2 IR model. MDC labelling was used to detect acidic vesicles, including autophagosomes formation [21, 37]. We found that autophagy flow in H9c2 cells exposed to 500 *μ*M PA rose in the first 24 hours and then declined. LC3II is considered to be an autophagosome marker in mammals, and higher LC3II/LC3I ratios indicate increased autophagy flow [26]. Experiments detecting GFP-LC3 puncta demonstrated that HCQ treatment significantly increased the accumulation of LC3II (designated as “LC3 net flux”) in each treatment after 24 h. These results demonstrate that PA activates excessive autophagy at its initial formation rather than blocking the fusion of autophagosomes with lysosomes. Excessive autophagy was also indicated by the increased LC3I to LC3II protein conversion, and the reduction in Atg protein (Atg3, Atg12-Atg5, and Atg16L1) levels in the 500 *μ*M PA groups. All of the above results led to a conclusion that 500 *μ*M PA induced excessive autophagy activation in H9c2 cells during IR development.

Phosphatidylinositol 3-kinase (PI3K)/Akt/mTOR pathway was a link between insulin resistance and autophagy. The two major insulin receptor substrates, IRS1 and IRS2, activate PI3K/Akt pathway [31], and the PI3K/Akt/mTOR pathway inhibits autophagy when it is activated [38]. It has been demonstrated that autophagy can be inhibited by activating mTOR [39]. In our study, the PI3Kp85 subunit was expressed at lower levels in the 500 *μ*M PA-treated group compared to the control cells, with the same tendency seen in the RAP group, suggesting that PI3K was inhibited by PA. The mRNA level of mTOR was increased by PA and PA + RAP, which might be due to the feedback of the protein levels for mTOR. Accumulating evidence shows that autophagy and apoptosis are executed through distinct signaling pathways, as autophagy is mainly mediated by Atg proteins [26] and apoptosis was mainly regulated by Bcl2 family protein and caspase family [40]. Yet overlapping signals are engaged in response to specific stimuli, as researchers showed that the crosstalk could be mediated by interactions between Beclin1 and Bcl2/Bcl-xl [41]. To explore the cytotoxicity of PA during the development of IR, we employed Annexin V-FITC/PI FCM to detect cell apoptosis [42, 43]. Our results indicate that 500 *μ*M PA also promoted apoptosis after treatment for 24 hours and more, while treatment for only 12 hours did not increase the rate of apoptosis. The protein levels for Bcl2 decreased dramatically in the PA and PA + RAP treated cells, compared with the controls, and the mRNA level for Beclin1 was increased with PA exposure. According to Wei et al. [27], rapid phosphorylation of Bcl2 may initially occur to promote cell survival by disrupting the Bcl2-Beclin1 complex and activating autophagy. When autophagy is no longer able to keep cells alive, the phosphorylated Bcl2 might then serve to inactivate its antiapoptotic function. Our findings suggest that the decreasing protein levels of Bcl2 might be another factor enhancing autophagy and apoptosis.

## 5. Conclusions

**Figure 6:**
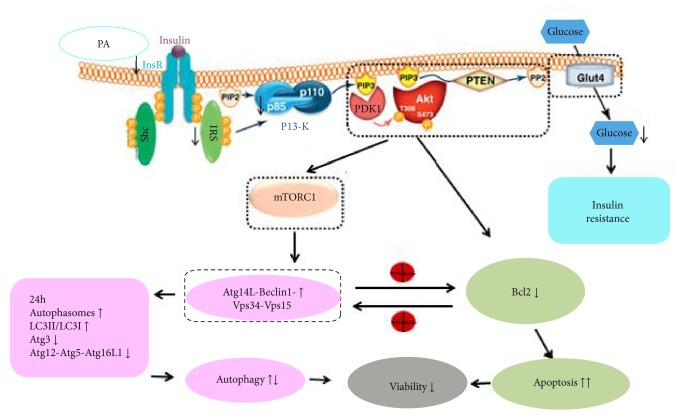
Relationship among PA-induced insulin resistance, autophagy, apoptosis, and inhibition of the PI3K/Akt/mTOR pathway. Insulin resistance was induced in H9c2 cells with 500 *μ*M PA for 24 h. During the development of IR, the numbers of autophagic acid vesicles increased during the first 24 hours and then decreased by 36 hours after PA treatment. Western blot analysis showed that the treatment of H9c2 cells with 500 *μ*M PA for 24 hours decreased the expression of Atg12-Atg5, Atg16L1, Atg3, and PI3Kp85. Annexin V/PI flow cytometry revealed that PA exposure for 24 hours increased the rate of apoptosis. The PI3K/Akt/mTOR pathway was inhibited after the PA treatment.

The current study showed that PA induces IR in H9c2 cells, and this process is accompanied by an excessive activation of autophagy and increases in apoptosis. PI3K/Akt/mTOR pathway is involved in this process; our conclusion scheme is illustrated in Figure 6. Our study implies that targeting the activation of autophagy may help treating insulin resistance in DCM progression.
